# RYR1 mutation screening 1992 – 2014: a genetic report on 22 years from the Würzburg MH unit

**DOI:** 10.1186/1471-2253-14-S1-A17

**Published:** 2014-08-18

**Authors:** Clemens R Müller, Judith Skirde, Edmund Hartung, Martin Anetseder, Yvonne Ricci, Gunilla Islander, Frank Schuster

**Affiliations:** 1 Department of Human Genetics, University of Würzburg, Würzburg, 97074, Germany; 2 Malignant Hyperthermia Investigation Unit, University Hospital Würzburg Würzburg, 97080, Germany; 3 Malignant Hyperthermia Investigation Unit, Lunds University Hospital, Lund, 22185, Sweden

## Background

The ryanodine receptor type 1 (RyR1) is one of the biggest known muscle proteins (4 x 565 kDa) acting as the major calcium release channel of skeletal muscle. A large number of mutations have been identified in the corresponding gene (*RYR1*) giving rise to a variety of clinical phenotypes: Malignant Hyperthermia susceptibility (MHS) and the group of congenital (core) myopathies.

## Materials and methods

In 1992, we began to screen MHS individual by PCR and restriction enzyme digestion for the few then known *RYR1* mutations. At around 1995, we switched to Sanger sequencing of selected exons (mutation hot-spots) and for a number of years, we analysed five exons in MHS (exons 17, 39, 40, 45, 46) and four in myopathic subjects (exons 95, 100, 101, 102). From 2002 on, we also included whole-gene sequencing of all 106 exons by Sanger or NGS technologies (starting from 2011). In total, we have screened 1.029 unrelated index cases. The great majority of malignant hyperthermia (MH) individuals had a positive in vitro contracture test (IVCT) result (MHS or MH equivocal (MHE)) or a likely clinical MH episode. Patients with congenital myopathies were referred on the basis of their clinical presentation.

## Results

Among the 305 individuals screened for the MHS hot-spot, we identified 16 different sequence variants in 46 individuals (15,8 %). In 41 patients, the mutation met the criteria for causality of the European MH group (EMHG) leaving only 5 individuals with unclassified variants.

In the myopathy group of 474 index cases, 37 variants were identified in 72 individuals by screening the “CCD-hot-spot” (15,2 %). In this group, only three mutations in 16 patients fulfilled the causality criteria of EMHG. Thus, for 56 individuals, the screening gave no clear-cut results.

Of note, all variants in the first two groups were single and of heterozygous genotype.

After switching to whole-gene analysis, 250 individuals of either clinical phenotype were analysed. At total of 101 variants were found in 90 patients (36 %). Of those, 54 variants in 71 subjects were single and heterozygous, while ten patients had two, five had three and three patients four variants each. Only 18 patients carried ten *bona fide* (EMHG) mutations.

The recurrency rates of mutations were low: of the 140 different variants observed, only 17 occurred more than once and only seven more than twice covering just 59 patients.

## Conclusions

Our longitudinal study reflects the known allelic heterogeneity of RYR1 mutations which poses a challenge for effective variant identification strategies to the present day. Although labor and cost have decreased with the advent of novel technologies, the problem of variant interpretation has increased proportional to the range and sensitivity of the screening methods. Since the vast majority of RYR1 variants result in amino acid substitutions their functional impact is not immediately evident. The causality criteria set by the EMHG are very strict in order to maximize clinical safety but this is in contrast to current practice in most other genetically heterogeneous disorders. In fact, the majority of “unclassified” variants identified in this study have been published in association with MHS and/or congenital myopathies and many of them meet all but one of the EMHG criteria. The reliability of computer algorithms for predicting the impact of amino acid substitutions on protein function has greatly improved but is still limited for large proteins like RyR1.

It is fair to say that the initial hope of genetic analysis eventually replacing the invasive IVCT has proven vain for the majority of cases. Similarly, a quick & cheap MH test which could be used the night before anesthesia will – if it ever comes true – most likely not be based on genetic analysis.

## Note

This abstract was awarded by the EMHG as best presentation in the field of experimental research.

